# A novel intron variant is associated with emerging *pfdhps* mutant haplotypes in West and Central African *Plasmodium falciparum*

**DOI:** 10.1016/j.ijpddr.2025.100611

**Published:** 2025-08-26

**Authors:** Emma Filtenborg Hocke, Helle Hansson, Ana Chopo-Pizarro, Adebanjo Jonathan Adegbola, Oluseye Bolaji, Peter Thelma Ngwa Niba, Innocent Mbulli Ali, Akindeh Nji, Wilfred Mbacham, Vito Baraka, Neema B. Kulaya, Gauthier Mesia Kahunu, Hypolite Muhindo Mavoko, Papy Mandoko Nkoli, Eric Mukomena Sompwe, Destin Mbongi, Patrick Mitashi, Valérie A. Bédia, Paterne A. Gnagne, Abibatou Konaté, William Yavo, Hervé Menan, Khalid Beshir, Andria Mousa, Colin J. Sutherland, Michael Alifrangis, Cally Roper

**Affiliations:** a Centre for Translational Medicine and Parasitology, Department of Immunology and Microbiology, University of Copenhagen, Denmark; b Department of Infectious Diseases, Copenhagen University Hospital, Copenhagen, Denmark; c Department of Infection Biology, Faculty of Infectious and Tropical Diseases, LSHTM, United Kingdom; d MRC Centre for Global Infectious Disease Analysis, Department of Infectious Disease Epidemiology, Imperial College London, London, W12 0BZ, United Kingdom; e Department of Pharmaceutical Chemistry, Faculty of Pharmacy, Obafemi Awolowo University, Ile-Ife, Nigeria; f The Biotechnology Centre, University of Yaounde I, Cameroon; g The Center for Health Implementation and Translational Research CHITRES, The Fobang Institutes, Yaounde, Cameroon; h National Institute for Medical Research, Tanga Centre, Tanzania; i Kilimanjaro Christian Medical University College KCMUCo, Moshi, Tanzania; j Department of Biochemistry, Faculty of Science, University of Dschang, Cameroon; k Department of Pharmacology and Therapeutics, University of Kinshasa, Kinshasa, Democratic Republic of the Congo; l Department of Tropical Medicine, University of Kinshasa, Democratic Republic of the Congo; m National Institute of Biomedical Research, Kinshasa, Democratic Republic of the Congo; n University of Lubumbashi, National Malaria Control Program, Ministry of Health, Democratic Republic of the Congo; o Centre Hospitalier Monkole, Kinshasa, Democratic Republic of the Congo; p Malaria Research and Control Center, National Public Health Institute, Abidjan, Côte d'Ivoire; q Department of Parasitology-Mycology, Training and Research Unit of Pharmaceutical and Biological Sciences, Abidjan, Côte d'Ivoire

**Keywords:** Plasmodium falciparum, Drug resistance, Sulfadoxine-pyrimethamine, Pfdhps, Evolution

## Abstract

Sulfadoxine-pyrimethamine plays a key role in *Plasmodium falciparum* chemoprevention across Africa, yet the protective efficacy of SP is undermined by mutations conferring resistance in the genes encoding dihydrofolate reductase (*pfdhfr*) and dihydropteroate synthase (*pfdhps*). The emergence and spread of the *pfdhps* 431V mutation suggests that this may confer resistance and be selected by drug use. Here, we report a non-coding mutation a548383t, which expands a di-nucleotide repeat in the first intron of *pfpppk-dhps*. The first intron and second exon of the *pfdhps* gene were analysed by target amplicon sequencing of 929 *P. falciparum*-positive blood samples from Nigeria, Cameroon, Tanzania, The Democratic Republic of Congo, and Côte d’Ivoire. The intron mutation was found in Nigeria, Côte d’Ivoire, and Cameroon in association with the 431V mutation. In particular, the intron mutation was most highly associated with the **VAG**K**GS** haplotype (OR = 211.7, P < 0.001), followed by the **VAG**KA**S** (OR = 39.2, P < 0.001), and **VAG**KAA (OR = 33.6, P < 0.001) haplotypes. Additionally, a reduced di-nucleotide repeat diversity was observed in 431V-positive variants. The intron variant is significantly associated with the 431V mutation which is consistent with previous reports of selective sweeps around **VAG**K**GS.** The association of the 548383t mutation with both **VAG**K**GS, VAG**KA**S** and **VAG**KAA might indicate these lineages either have a common ancestor or that the intron variant 548383t has a functional association with 431V. More research is needed to determine if the association is simply genetic hitchhiking, or if the intron variant confers a phenotypic advantage.

## Highlights

• The *pfdhps* 431V substitution has recently emerged in West Africa and may confer resistance to sulfadoxine-pyrimethamine.
• We report a new non-coding mutation a548383t in the first intron of *pfdhps*, which expands a di-nucleotide repeat.
• The intron variant was observed in Nigeria, Côte d’Ivoire and Cameroon significantly linked with 431V.
• Reduced di-nucleotide repeat diversity was associated with the 431V and intron variant.

## Graphical abstract

**Figure:**
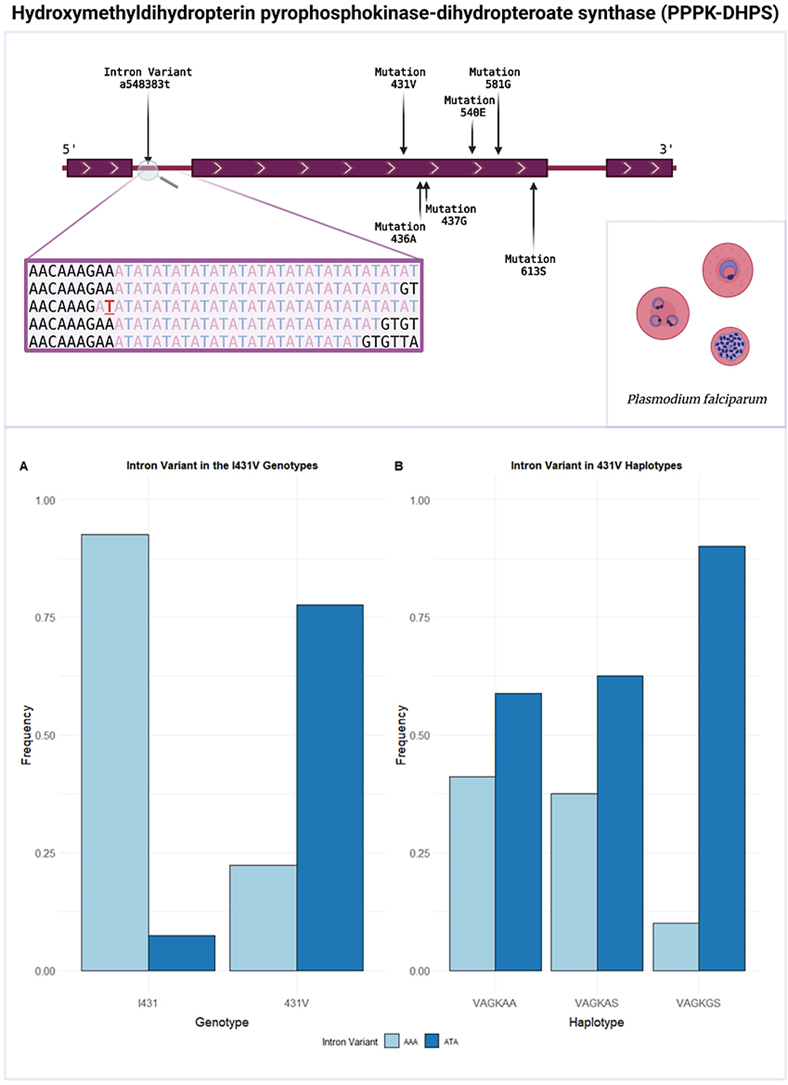

## 1 Introduction

*Plasmodium falciparum* (*Pf*) malaria is a global health concern causing a high rate of morbidity and mortality worldwide, particularly in sub-Saharan Africa, where an estimated 249 million cases and 608,000 deaths were reported for 2022 (WHO, 2023a). Even with the recent deployment of vaccines, the preventive measures against malaria still rely heavily upon chemoprevention (WHO, 2023a) and Sulfadoxine-pyrimethamine (SP) plays a pivotal role in this. Specifically, SP is used in Intermittent Preventive Treatment in Pregnancy (IPTp), Seasonal Malaria Chemoprevention (SMC; in combination with amodiaquine) in young children residing in areas with seasonal transmission, and Perennial Malaria Chemoprevention (PMC) in infants (Baba et al., 2020; WHO, 2023b). Resistance to SP threatens the protective efficacy of these interventions (WHO, 2023b), and is caused by the accumulation of point mutations in the *P. falciparum* dihydrofolate reductase (*pfdhfr*) and dihydropteroate synthase (*pfdhps*) genes (reviewed in (Venkatesan et al., 2013). Higher numbers of mutations at these two loci are associated with increased SP treatment failure (Kublin et al., 2002). The genotype comprising *pfdhfr* (51I, 59R, and 108N) and *pfdhps* (437G) mutant alleles is linked with partial resistance and found throughout West and Central Africa. The genotype made up of *pfdhfr* (51I, 59R, and 108N) with *pfdhps* (437G and 540E) mutant alleles is associated with SP treatment failure (Kublin et al., 2002), and further combination with additional variants in *pfdhfr* (164L) and/or *pfdhps* (581G and 613S) confers an even higher level of tolerance to SP (Naidoo and Roper, 2013; Triglia et al., 1997).

In East Africa, where the *pfdhps* mutations 437G+540E are highly prevalent, duration of SP protection is shorter than in West and Central Africa (Mousa et al., 2025). Studies on the protective efficacy of IPTp have found that SP still provided protection in areas where the prevalence of 437G+540E was >90 %, but that benefit was lost in areas where the *pfdhps* 581G was also present >10 % (Chico et al., 2015; Eijk et al., 2019).

In West Africa, parasite populations are generally regarded as only partially resistant to SP due to the absence of the 540E mutation. However, the emergence of a novel mutation, namely the 431V, is a cause for concern. First described in 2009 (Sutherland et al., 2009), the 431V mutation has been shown by crystal structure modelling to have the potential to reduce the effectiveness of sulfadoxine (Oguike et al., 2016). The 431V was first reported in Nigeria where it was seen in association with various combinations of 436A, 437G, 581G, and 613S mutations (Adegbola et al., 2023; Oguike et al., 2016; Sutherland et al., 2009). It has since been reported in Cameroon (Chauvin et al., 2015; Guémas et al., 2023; L'Episcopia et al., 2021), Benin (Svigel et al., 2021), Chad (Beshir et al., 2023), DRC (Guémas et al., 2023; Nkoli Mandoko et al., 2018), Guinea Bissau (Nag et al., 2017), Guinea-Conakry, Mali and Niger (Baba et al., 2020), Gabon, Central African Republic, and Republic of Congo (Guémas et al., 2023). Analysis of microsatellite markers in the *pfdhps* flanking region has indicated a recent selection of *pfdhps* alleles carrying the 431V mutant in isolates from Cameroon, Central African Republic, Democratic Republic of Congo, Gabon, Nigeria, and the Republic of Congo (Guémas et al., 2023).

Microsatellites are a common feature of the *P. falciparum* genome, often characterized by short tandem repeats of (TA)^n^ or (T or A)^n^ sequences repeated 10–30 times (Ferdig and Su, 2000). Microsatellite length variants are informative markers used in *P. falciparum* population genetics (Orjuela-Sánchez et al., 2013) and can serve as valuable tools for tracking the dispersal of resistance mutant lineages, as well as for studying the evolution, migration, and interrelatedness of *P. falciparum* strains (Kanai et al., 2022; Wootton et al., 2002). Analysis of microsatellites located 0.8 kb and 4.3 kb from *pfdhps* revealed that **VAG**K**GS** alleles from all countries share a single common origin (Guémas et al., 2023). Interestingly, the same study showed that microsatellite haplotypes associated with **VAG**KAA have a different origin to **VAG**K**GS** (Guémas et al., 2023), while **VAG**KA**S** alleles were split**;** 75 % shared the **VAG**KAA microsatellite haplotype and 25 % shared the **VAG**K**GS** flanking haplotype (Guémas et al., 2023).

Using next-generation sequencing of parasite DNA, this study aimed to explore the geographical extent across five African countries of a novel mutation, a548383t in the first intron of *pfdhps*, described here for the first time. Furthermore, the study explored whether there is evidence of an association between a548383t and haplotypes with or without the 431V, and to explore whether the length of the di-nucleotide repeat is linked to each haplotype.

## 2 Materials and methods

### 2.1 Sample selection

#### 2.1.1 Samples selected for initial sequence analysis

The a548383t intron mutation was first observed in this study from *pfdhps* sequences in the *Pf7* data release at Malaria Gen (Ahouidi et al., 2021). To investigate further, primer pairs were designed around the first intron of *pfdhps* where the variant is located. These primers were tested on a subset of samples with known *pfdhps* haplotype sequences selected from previously published studies in Nigeria (samples obtained from Ile-Ife, a town in Southwest Nigeria collected from 2020 to 2021) (n = 130) (see (Adegbola et al., 2023)) and Cameroon (collected in various sites in Yaounde in 2014–2020) (n = 46) (see (Niba et al., 2023)). The subset of samples was selected on the basis of having a good representation of both the 431V mutation and wildtype I431.

#### 2.1.2 Field survey samples

To assess the field prevalence of the intron variant and associated *pfdhps* haplotypes *P. falciparum* positive samples from surveys in four Countries were analysed. In Cameroon dried blood spots collected from symptomatic children and adults with confirmed malaria infection attending public health facilities between 2018 and 2022 (n = 704).

*P. falciparum* positive samples from symptomatic children aged 6–59 months attending health facilities in in 2020–2021 at four sites in the equatorial zone of The Democratic Republic of Congo; Rutshuru, Kabondo, Mikalayi, and Kapalowe, (see (Mesia Kahunu et al., 2024)).

Clinical samples from Côte d’Ivoire with unknown *pfdhps* haplotypes were collected in 2020 from malaria patients in Abengourou, San Pedro, and Abidjan (N = 319).

*P. falciparum* samples from Tanzania (N = 92), originated from cross-sectional surveys of all age groups in the Tanga region of Northeastern Tanzania, during peak malaria transmission in 2022.

### 2.2 Ethics approval

Ethical approval for collection and analysis of Nigerian samples during 2020–2021 (see (Adegbola et al., 2023)) was obtained from the Health Research and Ethics Committee (HREC) of the Obafemi Awolowo University Teaching Hospitals Complex, Ile-Ife (Protocol Number-ERC/2020/02/24).

Samples from a study from Yaounde Cameroon during 2014–2020 (Niba et al., 2023) received ethical clearance from the Cameroon National Ethics Committee (N 2013/11/372/L/CNERSH/SP), and administrative approval from the Ministry of Public Health, Yaounde, Cameroon.

Field sample collections in Cameroon 2018–2022 received ethical clearance from the National Ethics Committee for Human Health Research (reference 2018/01/962/CE/CNERSH/SP) and from the Cameroon Baptist Convention Health Services Institutional Review Board (number IRB1029-38).

Samples from DRC were collected as part of a therapeutic efficacy study in 2020–2021 see (Mesia Kahunu et al., 2024) with Ethical approval by The Democratic Republic of Congo Ethics Committee of the School of Public Health of the University of Kinshasa *Reference No ESP/CE/69/2020* and registered at www.ClinicalTrials.gov, registration number NCT0461852.

Tanzanian cross-sectional survey samples were collected in 2022 under a protocol which received ethical clearance from the Medical Research Coordinating Committee of the National Institute for Medical Research (NIMR-MRCC) with reference NIMR/HQ/R.8a/Vol.IX/3640.

Samples from Côte d’Ivoire were collected in Abengourou and San Pedro as part of a study with ethical approval from National Ethics Committee for Science and Health, (CNESVS) reference 149-20MSHP/CNEVS-km. Samples collected in Abidjan were part of a study with ethical approval from National Ethics Committee for Science and Health, (CNESVS) reference 081-20/MSHP/CNESVS-kp.

### 2.3 Amplification of *pfdhps* fragments

**Fig. 1:**
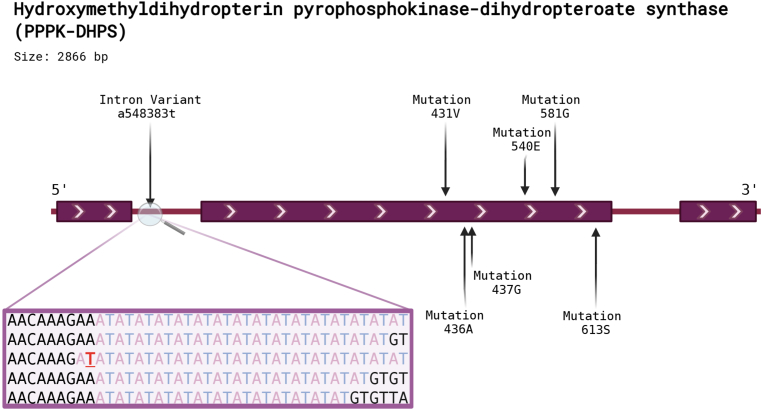
Illustrated representation of the intron variant at position 548383 in the first intron of the *pppk-dhps* gene expanding a microsatellite. The red letter T indicates the 548383t mutation. The alignment demonstrates the observed variation in the microsatellite, with the microsatellite region highlighted in light blue and light red color. Arrows denote the positions of resistance mutations 431V, 436A, 437G, 540E, 581G, and 613S located in exon 2. Created with BioRender.com.

The location of the intronic mutation within the di-nucleotide repeat in the first intron of *pfdhps*, upstream from the SP resistance-associated mutations, is shown in Fig. 1. Two different amplicons were sequenced, the first, covering the whole first intronic region (intron length, 176 bp) and the second, covering the exon 2, the site of known *pfdhps* mutations at codons 431, 436, 437, 540, 581 and 613 which code for amino acid changes associated with SP resistance.

Both regions of the *pfdhps* gene were amplified by nested PCRs on extracted DNA samples: Intron 1 was amplified using primers designed specifically for this study and exon 2 containing the drug resistance mutations of *pfdhps* was amplified using previously published primers and conditions (Nag et al., 2017) Primers are given in full in Supplementary Table 1. Cycling conditions for the outer PCR (intron 1) consisted of an initial denaturation of 95 °C for 15 min followed by 40 cycles of denaturation at 95 °C for 30 s, annealing at 53 °C for 45 s, and elongation at 68 °C for 1 min and 30 s, then a final elongation of 68 °C for 5 min. Cycling conditions for the nested PCR had an initial denaturation of 95 °C for 15 min followed by 35 cycles of denaturation of 95 °C for 30 s, annealing of 58 °C for 45 s, and elongation of 68 °C for 1 min and 30 s, followed by a final elongation step of 68 °C for 5 min.

### 2.4 Target amplicon sequencing of *pfdhps* regions

For sequencing of the *pfdhps* intron 1 and exon 2, samples were dual indexed with non-annealing overhangs in the nested PCR, and specific 8-base pair sequences were attached in an index PCR step for unique barcoding of each sample as described previously (Nag et al., 2017). After the index step, library preparation consisted of bead purification and dilution to a final concentration of 4 nM. A 5 % PhiX library was spiked in (Illumina, California, United States), to counteract the low nucleotide diversity caused by the high AT content in the *P. falciparum* genome. Sequencing by synthesis was then performed using the Illumina MiSeq V3 kit (Illumina, California, United States).

### 2.5 Quality control

For initial quality control, sequences were handled using the online software Galaxy (https://usegalaxy.org/). The FastQ files were quality-controlled using FastQC (Andrews) and the QC results were then aggregated using MultiQC (Ewels et al., 2016). Sequences were then trimmed using Trimmomatic (Bolger et al., 2014) with the following parameters: average Phred across 4 bases had to be above 20 (base-calling probability above 99). The trimmed sequences were then combined using the concatenate datasets function in Galaxy.

### 2.6 Analysis of *pfdhps* resistance-associated markers

After the quality control step, an investigation of the known *pfdhps* SNP was performed. The trimmed and combined fastq files were aligned to the 3D7 reference genome (Gardner et al., 2002) using *assimpler* (Leekitcharoenphon et al., 2014), a Python program to compare reads with a custom database (Nag et al., 2017). The following conditions were further applied: only SNPs with over 50 reads were accepted, and a cut-off to exclude mixed infection was set. Infections were classified as mixed if the minor variant was greater than 25 %. So for example if a SNP in a particular sample had 74 % of reads in base A and 26 % of reads in base T, it was considered a mixed infection with the genotype A/T. Mixed infections were excluded from the analysis of associations. (Table 1).

**Table 1:** Countries, regions and number of samples included for targeted *pfdhps* NGS analysis.

Country	Region(s)	Year of collection	N samples - collected	N samples sequencing positive^a^	Full Haplotypes (excl. mixed) spanning *pfdhps* and a548383t	Prevalence 431V from sequencing positive samples N (%)	Prevalence 548383t from sequencing positive samples N (%)	Study	Selection premise
Nigeria	Ife-Ife	2020–2021	130	57	42	23 (40.4 %)	24 (42.1 %)	Adegbola et al. (2023)	Preselected samples for initial analysis
Cameroon	Yaounde	2014–2022	46	43	32	10 (31.3 %)	7 (16.3 %)	Niba et al. (2023)	Preselected samples for initial analysis

Cameroon	Adamoua Bertoua Garoua Maroua Ngounso	2018–2022	681	350	237	59 (16.8 %)	79 (22.6 %)	This study	Field survey samples
Tanzania	Tanga	2022	92	30	27	0 (0 %)	0 (0 %)	This study	Field survey samples
DRC	Rutshuru, Kabonda, Mikalayi, Kapalowe	2020–2021	184	159	120	0 (0 %)	0 (0 %)	Mesia Kahunu et al. (2024)	Field survey samples
Côte d’Ivoire	Abengourou, San Pedro, Abidjan	2020	319	290	226	5 (1.7 %)	1 (0.34 %)	This study	Field survey samples
Total			1452	929	684	97	111		

a Positive sequencing samples indicate the sample were positive in both codon I431V and a548383t position in the microsatellite, but not necessarily in other resistance codons.

### 2.7 Consensus sequence construction of the a548383t intron region

For closer inspection of the diversity of the first intron of *pfdhps,* a consensus sequence approach was used. The trimmed FastQ files from the Quality Control step were aligned using the Rsubread package to a *pfdhps* reference gene (Gene ID: 2655294, retrieved from https://www.ncbi.nlm.nih.gov/) (Liao et al., 2019). A consensus sequence was then constructed using quasitools, with a majority base consensus (quasitools). Sequences were visualized and inspected using MEGA X (Tamura et al., 2021) and sequences were aligned using the MUSCLE algorithm (Edgar, 2004). Additionally, results were compared using the Malaria Profiler software (https://bioinformatics.lshtm.ac.uk/malaria-profiler/) (Phelan et al., 2023).

### 2.8 Statistical analysis

Statistical analyses were conducted using R Studio (Team, 2022), with a significance level set at 5 %. Chi-square tests were employed to evaluate significant differences between qualitative variables of genotypes. Additionally, a univariable logistic regression model was fitted to assess the association between haplotypes, and intron mutation status, using the wild type I431 haplotype as the reference category. Crude odds ratios and their 95 % confidence intervals (CIs) were subsequently estimated.

## 3 Results

### 3.1 *P. falciparum* positive samples available for analysis

In total, 929 *P. falciparum* positive samples from Tanzania, DRC, Nigeria, Côte d’Ivoire, and Cameroon were included in the study (Table 1). Following exclusion of mixed infections from the analysis, a total of 764 samples had a genotype profile with sequence for codon I431V and the intron variant, and of these 684 samples had a full genotype profile covering both the intron and codons 431,436,437,540,581 and 613 of the exon and could be used for analysis of association between intron variant and *pfdhps* haplotype.

### 3.2 Prevalence of the intron variant a548383t and the *pfdhps* 431 mutation alone

The prevalence of the 431V mutation and the intron variant are shown for each sample set in Table 1. The intron variant 548383t was seen only in Nigeria, Cameroon and Cote d’Ivoire, all settings where the 431V mutation was also prevalent. Neither the 431V mutation nor intron variant were seen in samples from DRC and Tanzania.

The 548383t mutation occurred in 111 out of the 929 positive samples. Out of these, 86 (77.5 %) originated from Cameroon, 24 (21.6 %) from Nigeria, and 1 (0.9 %) from Côte d’Ivoire.

**Fig. 2:**
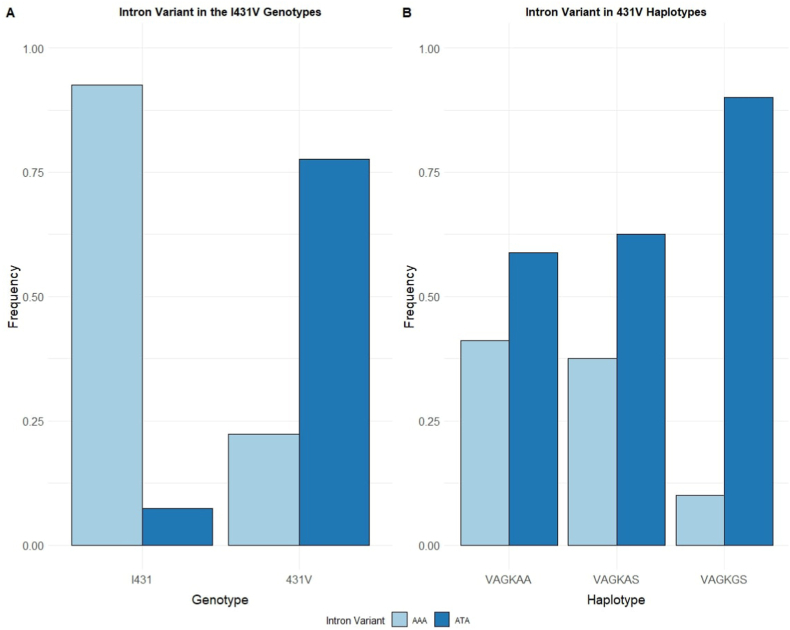
**Distribution of the a548383t intron variant in I431V containing -genotypes and 431V-containing haplotypes in samples from Nigeria and Cameroon**. A) Frequency of the *pfdhps* a548383t intron variant in I431V-containing genotypes. B) Frequency of the intron variant observed in 431V-containing haplotypes (codons 431-436-437-540-581-613). The samples included in this study were selected based on a combination of previously identified 431V haplotypes as reported from previous published studies, and novel population samples. Consequently, the plots should not be interpreted as representative of geographical prevalence of the 431V variant.

In samples from Cameroon, Nigeria and Côte d’Ivoire, the prevalence of 431V was 17.55 % (69/393), 40.4 % (23/57) and 1.7 % (5/290), respectively. Dividing the samples into *pfdhps*-I431 and 431V genotypes, the intron variant was found to be significantly associated with the 431V mutation (OR = 43.5, 95 % CI [24.3, 77.7], χ^2^ = 3.5, P < 0.001) (Fig. 2A).

### 3.3 The intron variant association with specific *pfdhps* 431V haplotypes

To further explore the relationship between the intron mutation and *pfdhps*-431V, combined haplotype profiles were analysed. A total of 92 *pfdhps* haplotype profiles containing the 431V were available for analysis. These were from Nigeria (n = 19), Côte d’Ivoire (n = 4), and Cameroon (n = 69). Three different 431V-containing haplotypes were observed at codons 431-436-437-540-581-613: **VAG**K**GS** (n = 50), **VAG**KAA (n = 34) and **VAG**KA**S** (n = 8), 90.0 % of the **VAG**K**GS** haplotypes (n = 45) harbored the intron mutation (OR = 211.7, 95 % CI [74.5, 594.1], P < 0.001), followed by 62.5 % of the **VAG**KA**S** haplotypes (n = 5, OR = 39.2, 95 % CI [8.7, 176.3], P < 0.001), and **VAG**KAA (58.8 %, n = 20, OR = 33,6, 95 % CI [14.8, 76.5], P < 0.001) (Fig. 2B).

Samples having the **VAG**K**GS** haplotype from Cameroon and Nigeria almost exclusively had the 548383t intron mutation, but interestingly, in Côte d’Ivoire, five samples with the 431V genotypes were uncovered, and four of them with the **VAG**K**GS** haplotype. None of the samples with the **VAG**K**GS** haplotype harbored the intron mutation, only the one sample with **VAG**KAA had the intron mutation.

### 3.4 Microsatellite diversity and *pfdhps* 431V

**Fig. 3:**
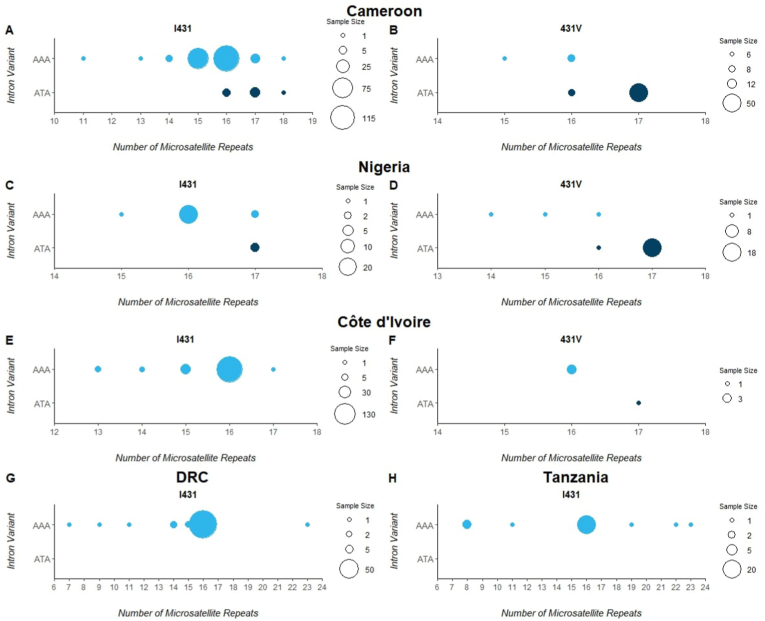
**Weighted scatterplot of microsatellite length in *pfdhps* showing counts of I431 wildtypes and 431V mutants and the intron mutant 548383t (ATA) or Wildtype (AAA) across Five African countries. A&B)** the I431V variants in Cameroon, **C&D)** the I431V variants in Nigeria, **E&F)** the I431V variants in Cõte d’Ivoire, **G)** the I431 wildtype in DRC. **H)** the I431 wildtype in Tanzania. The samples included in this study were selected based on a combination of previously identified 431V haplotypes as reported from previous published studies, and novel population samples. Consequently, the plots should not be interpreted as representative of geographical prevalence of the 431V variant.

The diversity in length of the di-nucleotide repeat region was measured in samples obtained from the five countries and this is summarized in Fig. 3, where repeat numbers in the microsatellite are shown on the x-axis in charts contrasting *pfdhps*-I431 vs *pfdhps* 431V in Cameroon (3A and 3B), Nigeria (3C and 3D) Cote D'Ivoire (3E and 3F). The presence/absence of intron mutation 548383t is shown on the y axis in Fig. 3). In Cameroon, the microsatellite length varied from 11 to 18 repeats, and in Côte d’Ivoire 13–17 repeats. In Tanzania, it ranged between 8 and 23 repeats, and in DRC between 7 and 23 repeats. Nigeria had the narrowest variability in the di-nucleotide repeat number, ranging between 14 and 17 repeats. In samples with both the 431V and the 548383t intron mutant alleles, a microsatellite length of 17 was common across all three countries, Cameroon (n = 53/61), Nigeria (n = 18/19) and in Côte d’Ivoire (n = 1/1). In samples with I431, microsatellite repeat length was variable with a microsatellite repeat number of 16 being the most common across all countries (n = 498/617).

### 3.5 Microsatellite diversity associated with all *pfdhps* mutant haplotypes

**Fig. 4:**
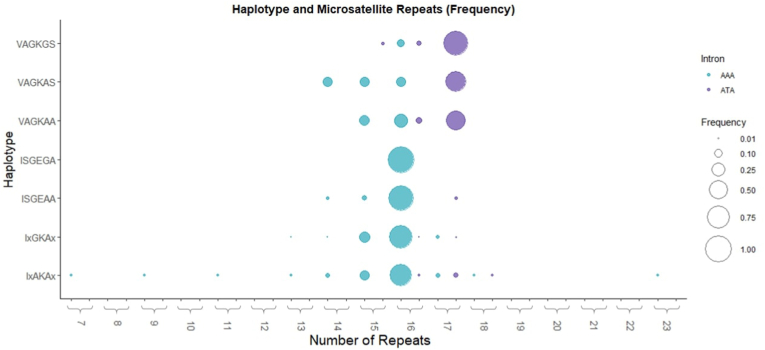
**Barplots showing frequencies in number of microsatellite repeats and whether the intron mutation is present (ATA) or the intron wildtype (AAA) for each *pfdhps* haplotype group.** Haplotypes containing an ‘x’ indicates that both the mutant and Wildtype at the codon position was grouped together for analysis. The samples included in this study were selected based on a combination of previously identified 431V haplotypes as reported from previous published studies, and novel population samples. Consequently, the plots should not be interpreted as representative of geographical prevalence of the 431V variant.

The di-nucleotide repeat association with *pfdhps* haplotypes in the five African countries was explored. Haplotypes made of *pfdhps* codons 431, 436, 437, 540, 581 and 613 respectively were grouped into 7 categories: **VAG**K**GS**, **VAG**KA**S**, **VAG**KAA, IxAKAx, Ix**G**KAx, IS**GE**AA, and IS**GEG**A (where ‘x’ indicates either S436 or 436A at codon 436, and either A613 or 613S in codon 613). In Fig. 4 we compare the di-nucleotide repeat polymorphism found in association with the 7 haplotype categories (presence of the intron variant is indicated by colour). The wild**-**type *pfdhps* haplotype IxAKAx had the highest diversity with di-nucleotide repeat number spanning 7–23, with 16 repeats being the most prevalent (n = 50/73 (68.6 %)). Similarly, the other I431-containing *pfdhps* haplotypes most commonly had 16 AT repeats. Low diversity in microsatellite repeat number was particularly evident for the triple mutant IS**GEG**A where all samples (n = 16) carried exactly 16 repeats. By contrast, for the 431V-containing *pfdhps* haplotypes, the majority had 17 di**-**nucleotide repeats, and carried the intron mutation. This was particularly evident for the **VAG**K**GS** haplotype where 43/50, (86.0 %) had repeat number of 17.

## 4 Discussion

Over the last two decades, reports of the emergence and spread of the *pfdhps*-431V mutation through West and Central Africa has increased. (Adegbola et al., 2023; Guémas et al., 2023; Oguike et al., 2016; Sutherland et al., 2009). Although an effect on resistance has not been verified *in vitro,* the rising number of reports raises the possibility that a possible high-grade resistance is spreading favoured by drug selection. A recent study found a significant overrepresentation of the **VAG**K**GS** haplotype among symptomatic malaria infections in pregnant women who had received SP (Cohen et al., 2023).

This study analysed the sequence of an intron fragment upstream of the *pfdhps* gene, and revealed association between the intron variant (548383t) and the *pfdhps*-431V. A strong association between the 548383t variant and the **VAG**K**GS** haplotype was observed and a weaker association with the **VAG**KAA haplotype, suggesting a nuanced evolutionary dynamic in the emergence of these genetic variations. A strong association between microsatellite repeat number and the **VAG**K**GS** haplotype further supports that drug selection pressure favoring the **VAG**K**GS** haplotype and the intron variant.

It is possible that the **VAG**K**GS** haplotype developed from the progenitor **VAG**KAA haplotype which carried the intron mutation. Guémas et al. (2023) investigated the genetic landscape surrounding the *pfdhps* locus and identified a common flanking region around *pfdhps* in 431V mutant haplotypes across several African countries indicating the 431V haplotypes emerged from a common ancestor (Guémas et al., 2023). In our study, it is noteworthy that **VAG**K**GS** samples from Côte d’Ivoire did not harbor the same intron mutation, perhaps indicating an independent origin of **VAG**K**GS** alleles into that country, although the sample size was limited (n = 4).

In association with wildtype *pfdhps* the microsatellite varied from 11 to 17 repeats in Nigeria, Côte d’Ivoire, and Cameroon or 7–23 repeats in DRC and Tanzania (Fig. 4). In association with more highly resistant *pfdhps* haplotypes the full intron sequence was generally conserved. When the adenine to thymine substitution occurs in the intron, the 548383 position becomes part of the upstream di-nucleotide repeat. It should be noted, with exception of the 548383t mutation happening upstream towards the 5'end that the microsatellite length only varied downstream (Fig. 1). Even though the vast majority of the samples with intron mutation with 17 repeats was found in 431V haplotypes, a few I431 wildtypes did also harbor the mutation. This could implicate that the mutated population with the 17 repeats served as the progenitor to the development of the 431V.

Interestingly, the East-African IS**GEG**A haplotypes had no intron mutant, but had a very fixed dinucleotide repeat length of 16 repeats instead of 17. This loss of microsatellite diversity flanking the *pfdhps* 540E and 581G haplotype has previously been observed. Pearce et al. investigated microsatellites flanking *pfdhps* and found a loss of diversity flanking *pfdhps* haplotypes carrying the 437G+540E mutations, identifying two independent origins of the IS**GE**AA in East Africa (Pearce et al., 2009). Another study by Alifrangis et al., investigating S**GEG**A haplotypes in Ethiopia, Tanzania, and Uganda (Alifrangis et al., 2014a) found reduced microsatellite diversity consistent with stronger drug selection pressure. Supporting these findings, McCollum et al., also documented the occurrence of a similar selective sweep in Kenya (McCollum et al., 2012).

In microsatellites flanking a locus under selection, a significant reduction in genetic diversity is frequently reported, contrasting with higher levels of diversity in the same loci flanking the wild-type sensitive allele of the same locus (Alifrangis et al., 2014; Guémas et al., 2023; Pearce et al., 2009). In this study, in samples obtained from Nigeria, low genetic diversity was observed in the microsatellite in samples the 431V and intron mutant were both present, and the length of the microsatellite was highly conserved, with the vast majority having a length of 17 repeats. Parasites from Cameroon also had low diversity in the intron and in the microsatellite. These two countries border one another, and that could explain the similarity. However, Nigerian samples were collected in the Osun state, which is far away from the Cameroonian border.

While microsatellites have mainly been described as neutral markers of diversity, some studies have found evidence of a role in gene transcription regulation through mechanisms such as alternative splicing (Bagshaw, 2017; Li, 2004). The intronic di-nucleotide repeat in *pfdhps* is conserved across several other *Plasmodium* species, but the expansion with the 548383t mutation has only been observed in *P. falciparum* (Supplementary Fig. 1). Expansion of microsatellites has been shown to block intron splicing, leading to retention of the intron in the mature mRNA (Sznajder et al., 2018).

Some studies have utilized diverse sequencing methods have provided evidence of alternative splicing occurring in *P. falciparum*, playing a vital role in gene regulation (Lee et al., 2021; Lu et al., 2007; Shaw et al., 2022; Yeoh et al., 2019). For example, a study by Shaw et al. uncovered as many as 12,495 novel isoforms in *P. falciparum*, with intron retention and alternative 5′ and 3′ ends being the most common forms of alternative splicing (Shaw et al., 2022). Similarly, a study conducted by Lee et al., a notably high occurrence of intron retention was observed, including within the second intron of the *dhps* gene, which had not been previously reported. Additionally, various splicing isoforms were identified in other genes within the folate pathway (Lee et al., 2021; Supplementary Table 2). However, while alternative splicing is a well-documented phenomenon in metazoans, its characterization in apicomplexans, such as *P. falciparum*, remains limited (Yeoh et al., 2019). The emergence and rapid dispersal of the novel variant could testify to a possible selective advantage conferred by the combination of the intron change and the I431V, A581G and A613S *pfdhps* mutations, the nature of which remains to be determined.

This study used both previously published data and newly collected samples. Certain 431V-containing haplotypes were drawn from prior studies, while other samples represented broader population studies. Consequently, the study does not offer a comparable view of the geographical prevalence of 431V haplotypes. Furthermore, the consensus sequences for the intron variant were created by merging all Illumina reads in a sample, a method that may obscure rare variants but provides a broader understanding of diversity at this locus.

Our study sheds light on the intricate relationship between the intron variant a548383t, the microsatellite length, and the emerging *pfdhps* mutant haplotypes with 431V. A strong association with the VAGKGS haplotype, as well as a weaker linkage to the VAGKAA haplotype, suggests linked emergence events followed by dispersal into Cameroon and Nigeria. Considering the role of SP in malaria prevention strategies, further research is needed to track continuing dispersal of 431V haplotypes, to investigate their potential impact on SP resistance and its consequences for the protective efficacy of mass interventions such as SMC and PMC.

## CRediT authorship contribution statement

**Emma Filtenborg Hocke:** Writing – review & editing, Writing – original draft, Visualization, Methodology, Investigation, Formal analysis, Data curation. **Helle Hansson:** Writing – review & editing, Methodology, Investigation. **Ana Chopo-Pizarro:** Writing – review & editing, Methodology, Data curation. **Adebanjo Jonathan Adegbola:** Writing – review & editing, Data curation. **Oluseye Bolaji:** Writing – review & editing, Data curation. **Peter Thelma Ngwa Niba:** Writing – review & editing, Data curation. **Innocent Mbulli Ali:** Writing – review & editing, Data curation. **Akindeh Nji:** Writing – review & editing, Data curation. **Wilfred Mbacham:** Writing – review & editing, Data curation. **Vito Baraka:** Writing – review & editing, Data curation. **Neema B. Kulaya:** Writing – review & editing, Data curation. **Gauthier Mesia Kahunu:** Writing – review & editing, Data curation. **Hypolite Muhindo Mavoko:** Writing – review & editing, Data curation. **Papy Mandoko Nkoli:** Writing – review & editing, Data curation. **Eric Mukomena Sompwe:** Writing – review & editing, Data curation. **Destin Mbongi:** Writing – review & editing, Data curation. **Patrick Mitashi:** Writing – original draft, Data curation. **Valérie A. Bédia:** Writing – review & editing, Data curation. **Paterne A. Gnagne:** Writing – review & editing, Formal analysis. **Abibatou Konaté:** Writing – review & editing, Data curation. **William Yavo:** Writing – original draft, Funding acquisition, Data curation. **Hervé Menan:** Writing – review & editing, Funding acquisition, Data curation. **Khalid Beshir:** Writing – review & editing, Data curation. **Andria Mousa:** Writing – review & editing, Visualization, Data curation. **Colin J. Sutherland:** Writing – review & editing, Supervision, Investigation, Data curation, Conceptualization. **Michael Alifrangis:** Writing – review & editing, Validation, Supervision, Investigation, Funding acquisition, Data curation, Conceptualization. **Cally Roper:** Writing – review & editing, Supervision, Investigation, Funding acquisition, Data curation, Conceptualization.

## Funding

This analysis was supported by funding from UNITAID as part of the Plus Project (Grant number 101150IC). The study collection of samples from Tanzania was supported by Danida Fellowship Centre (DFC) under The Ministry of Foreign Affairs of Denmark through the Predicting vector-borne disease epidemics: Dissemination of risk forecasting using District Health Information Software2 in Tanzania (preVBD) project (DFC file no. 19-02-KU).

Collection of samples from Nigeria was originally supported by an EDCTP2 Preparatory Fellowship (TMA2018PF-2537) to Adebanjo Adegbola. Collection of samples from Cameroon was originally funded in whole by the Wellcome Trust (grant # 107741/A/15/Z) and the UK Foreign Commonwealth and Development Office, with support from the Developing Excellence in Leadership, Training and Science in Africa (DELTAS Africa) program.

## Conflict of interest

The authors declare no conflict of interest.
