# Supplementary material for: A novel intron variant is associated with emerging pfdhps mutant haplotypes in West and Central African Plasmodium falciparum
Source: Int J Parasitol Drugs Drug Resist. 2025 Aug 26;29:100611. doi: 10.1016/j.ijpddr.2025.100611 (PMC12410353; doi:10.1016/j.ijpddr.2025.100611)
Supplement: Multimedia component 1 [file mmc1.docx]

**Supplementary**

**Supplementary table 1**: Out and nested primers used to amplify the microsatellite intron region and exon2 in *pfdhps.*

*Primers published by Nag et al., 2017(Nag et al., 2017)

| **Primer Name** | **Primer Sequence (5’-3’)** | **Amplicon size** | **Anneal. Tm** |
| --- | --- | --- | --- |
| IntronMut_Out_Fw | GGAACAAATGATAGAAGAAACGC | ~358 bp | 53 °C |
| IntronMut_Out_Rv | CATCATATATACGACAATCCTTG |  |  |
| IntronMut_Nest_Fw | CTGCTCTGCACCTTGTCGAAA | ~262 bp | 58 °C |
| IntronMut_Nest_Rv | GGAACGGTTTCATACAAGTAGGACG |  |  |
| Exon2Mut_Out_Fw* | ACAAATATGTGAGTAGGATGAAAGAACAA | ~884 bp | 52 °C |
| Exon2Mut_Out_Rv* | CATCCAATTGTGTGATTTGTCCACAATAT |  | 52 °C |
| Exon2Mut_Nest_Fw* | GGAATATTAAATGTTAATTATGATTCT | ~692 bp | 52 °C |
| Exon2Mut_Nest_Rv* | ATTACAACATTTTGATCATTCATGCA |  | 52 °C |

**Supplementary table 2:** Count of the resulting *pfdhps* haplotypes included for further haplotype analysis in this study. Haplotypes containing an ‘x’ indicates that both the mutant and Wildtype at the codon position was grouped together for analysis.

| **Haplotype** | **Count** |
| --- | --- |
| IxAKAx | 96 |
| IxGKAx | 428 |
| ISGEAA | 52 |
| ISGEGA | 16 |
| VAGKAA | 34 |
| VAGKAS | 8 |
| VAGKGS | 50 |


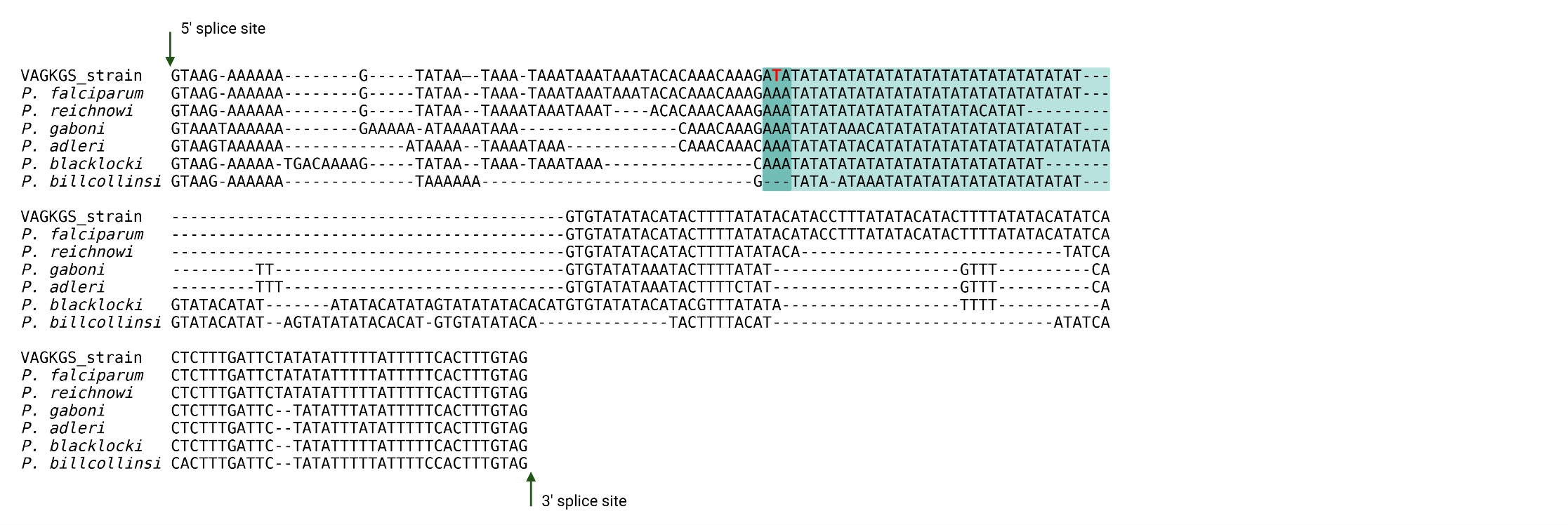


**Supplementary Figure 1.** Alignment of the first intron of the dhps gene across various Plasmodium species, including the P. falciparum VAGKGS strain. The light turquoise highlights the microsatellite region, while the dark turquoise marks the site of microsatellite expansion associated with the intron mutation. The 548383t intron mutation, which involves an A-to-T substitution, is indicated in bright red. Created with BioRender.com
